# Resisting and challenging stigma in Uganda: the role of support groups of people living with HIV

**DOI:** 10.7448/IAS.16.3.18636

**Published:** 2013-11-13

**Authors:** Gitau Mburu, Mala Ram, Morten Skovdal, David Bitira, Ian Hodgson, Grace W Mwai, Christine Stegling, Janet Seeley

**Affiliations:** 1 International HIV/AIDS Alliance, Hove, UK; 2 Division of Health Research, Lancaster University, Lancaster, UK; 3 Institute of Social Psychology, London School of Economics and Political Science, London, UK; 4 Community Health Alliance Uganda, Kampala, Uganda; 5 Centre for Global Health, Trinity College Dublin, Dublin, Ireland; 6 Division of Medical Education, Brighton and Sussex Medical School, University of Brighton, Brighton, UK; 7 School of International Development, University of East Anglia, Norwich, UK; 8 MRC UVRI Uganda Research Unit on AIDS, Entebbe, Uganda

**Keywords:** HIV, stigma, Uganda, Africa

## Abstract

**Introduction:**

Global scale up of antiretroviral therapy is changing the context of HIV-related stigma. However, stigma remains an ongoing concern in many countries. Groups of people living with HIV can contribute to the reduction of stigma. However, the pathways through which they do so are not well understood.

**Methods:**

This paper utilizes data from a qualitative study exploring the impact of networked groups of people living with HIV in Jinja and Mbale districts of Uganda. Participants were people living with HIV (*n=*40), members of their households (*n=*10) and their health service providers (*n=*15). Data were collected via interviews and focus group discussions in 2010, and analyzed inductively to extract key themes related to the approaches and outcomes of the groups’ anti-stigma activities.

**Results:**

Study participants reported that HIV stigma in their communities had declined as a result of the collective activities of groups of people living with HIV. However, they believed that stigma remained an ongoing challenge. Gender, family relationships, social and economic factors emerged as important drivers of stigma. Challenging stigma collectively transcended individual experiences and united people living with HIV in a process of social renegotiation to achieve change. Groups of people living with HIV provided peer support and improved the confidence of their members, which ultimately reduced self-stigma and improved their ability to deal with external stigma when it was encountered.

**Conclusions:**

Antiretroviral therapy and group-based approaches in the delivery of HIV services are opening up new avenues for the collective participation of people living with HIV to challenge HIV stigma and act as agents of social change. Interventions for reducing HIV stigma should be expanded beyond those that aim to increase the resilience and coping mechanisms of individuals, to those that build the capacity of groups to collectively cope with and challenge HIV stigma. Such interventions should be gender sensitive and should respond to contextual social, economic and structural factors that drive stigma.

## Introduction

HIV stigma is a clearly documented obstacle to HIV testing [1, 2], disclosure of HIV status [3, 4], uptake of antiretroviral therapy and retention in care [5]. HIV stigma can also aggravate mental health problems [6, 7] and significantly reduce the quality of life of people living with HIV [8]. There is therefore an urgent need to de-stigmatize HIV.

HIV stigma exists worldwide, and common drivers and manifestations of HIV stigma are recognized across different settings [9]. At the same time, the extent to which HIV stigma is experienced by people living with HIV varies considerably within and across different contexts. Experiences of HIV stigma may be shaped, for instance, by underlying stigmatization of specific behaviours such as sex work and injecting drug use, as well as by individual resilience [10].

There is a wide body of literature exploring HIV stigma, which is now recognized as a complex multidimensional phenomenon [5, 11, 12]. As such, it has proved challenging to define. Deacon *et al*. [12, p. 19] identify core elements of HIV stigma when they propose defining it as “an ideology that claims that people with a specific disease are different from ‘normal’ society, more than simply through their infection with a disease agent,” and also as a “social process by which people use shared social representations to distance themselves and their in-group from the risk of contracting a disease.” An exploration of this social process shows that HIV stigma is often influenced by the contribution an individual makes to society, that is, whether he or she is regarded as a drain on communal resources [13, 14].

Such material symbolism of stigma is pertinent as more people living with HIV enrol for treatment, live longer and gain employment [15, 16]. Widespread availability of treatment has been associated with an improved or so called “Lazarus” health outcomes, regained self-esteem [11], improved life expectancy [17] and reduced HIV stigma, for instance in Uganda and Botswana [18, 19]. These findings, which appear to confirm prior predictions that antiretroviral therapy could reduce HIV stigma [13], have led some researchers to question the extent to which HIV stigma persists in countries such as Uganda and its relevance to future HIV programming [20].

In a review of interventions targeting HIV-related stigma, Brown *et al*. [21] describe a conceptual framework that includes four types of approaches for de-stigmatizing HIV: first, information-based approaches, such as brochures; second, skills-building activities and other hands-on learning strategies that counter negative attitudes; third, counselling approaches; and fourth, contact with people living with HIV, for instance through testimonials and interaction with the general public.

In this paper, we consider the fourth approach, that is, pathways through which contact between people living with HIV and their communities could contribute to de-stigmatizing HIV. In particular, we explore the extent to which these interactions are influenced by the collective efficacy or resistance of people living with HIV, that is, the extent to which they take action to change their own circumstances [22].

This is important given that recent studies conducted in Zimbabwe, Tanzania and Botswana have shown that simply increasing the availability of antiretroviral treatment and counselling may not, on its own, be sufficient to reduce HIV stigma. Rather, in order to have an impact on stigma, antiretroviral therapy should be coupled with strategies that enable people living with HIV to better cope with and resist stigma, such as peer support groups [23, 24]. In this paper, we build on these findings by exploring how people living with HIV in Uganda contribute collectively to countering stigma. Based on recommendations from Brown *et al*. [21], we examine how groups of people living with HIV can nurture a collective efficacy that protects their members from the negative effects of stigma, while at the same time contributing to the de-stigmatization of HIV. Our focus is on “groups” as the unit of analysis rather than individual-level support, which is already well documented in Uganda, for instance in relation to The AIDS Support Organisation (TASO) model [25].

## Methods

### Setting

Data presented in this paper were collected as part of a qualitative study documenting the model and activities of networked groups of people living with HIV in Uganda, whose main findings are reported elsewhere [26, 27]. This paper focuses specifically on stigma reduction, based on previously unpublished data. Data were collected between June and October 2010 in Uganda's Mbale and Jinja districts, where the International HIV/AIDS Alliance had implemented a community-based HIV initiative known as the “Networks project” during the preceding four years, whose aim was to increase access to a comprehensive continuum of HIV services.

### Intervention

Central to the Networks project was the concept of meaningful involvement of groups of people living with HIV, which empowered them to be engaged as *partners* in the delivery of HIV services, as opposed to being *passive recipients* of services [28]. This was achieved through three approaches: first, mapping and supporting 750 existing groups of people living with HIV to organize themselves into a network of 120 larger sub-national clusters; second, training the groups on comprehensive HIV prevention and care, record keeping, income generation, advocacy and financial and general project management; and third, implementing community-based HIV prevention, care and treatment referral activities with the groups as partners, as described in detail elsewhere [27]. These groups were functional in 40 districts, with a total membership of more than 40,000 people living with HIV [27, 28].

### Group activities

Groups of people living with HIV mobilized their peers; provided community education; acted as patient ushers at HIV clinics; visited homes of people living with HIV; counselled household members on how to care for people living with HIV without prejudice; and performed HIV sensitization campaigns aimed at their communities. All of these activities were intended to increase HIV service uptake, but some may also have contributed to countering HIV stigma. Following the implementation of the project, this qualitative study was performed to explore processes leading to change, using two districts that represent diverse rural (Mbale) and urban (Jinja) settings.

### Participants

This paper, which focuses on HIV stigma, includes data from all 65 participants in the larger qualitative study: 40 people living with HIV (*n=*40), members of their households (*n=*10) and their health service providers (*n=*15), who were initially selected based on their previous involvement with the Networks project and their willingness to participate. Diverse participants were selected to enable triangulation of findings and to ensure that a wide range of perspectives would be captured [29], given that perceptions of HIV stigma in Uganda can differ between health service providers and family members [20]. A total of 25 study participants provided interviews, and the other 40 participants contributed to focus group discussions (Table 1).

**Table 1:** Study participants and methodology

	Interviews		Focus group discussions	
Population	People living with HIV*	Key informants**	People living with HIV*	Members of households with people with HIV
Sample size	10	15	3 sessions; *n=*30	1 session; *n=*10
Location	Jinja, Mbale	Jinja, Mbale	Jinja, Mbale	Jinja

* Examples of groups of people living with HIV from which participants were selected include Jinja People Living with HIV/AIDS Drama Group, Positive Men's Union, WIDE, Abatwogerera, NAKOLO, Khulirire Adwela, Mukwano Women's Association and Food Security TASO Group.

** Key informants included district health officers, district HIV focal persons, district AIDS coordinators, community leaders, medical superintendents of district hospitals, antiretroviral therapy clinic supervisors and leaders of groups of people living with HIV.

### Data collection

Interview guides and topics for the focus group discussions were developed in reference to existing gaps in the literature and the study objectives. These included exploring why people living with HIV formed (or joined) groups with others; how groups related to each other; how groups facilitated disclosure and visibility for people living with HIV; and how group activities influenced stigma and uptake of services (*see* Additional file 1 for topic guides). The tools were validated during a pilot phase that took into account the contextual environment of the study setting. These tools were then translated into Luganda and Lusoga for use when participants preferred to be interviewed in local languages instead of English. In these instances, a researcher who could speak that language conducted the interviews or focus group discussions. Researchers back-translated the local versions of the study tools to ensure that the meaning of the questions had not been altered. Interviews lasted 25–50 minutes, while focus group discussions lasted 45–60 minutes. Interviews and focus group discussions were conducted by researchers who were trained on ethical study conduct. Interviews and focus group discussions were audio recorded and transcribed. Data in Luganda and Lusoga were translated into English.

### Data analysis

Data were reviewed and all text segments subjected to a thematic analysis using QSR International's NVivo 7 [30], based on the initial study questions. These questions focused on the role of groups of people living with HIV in disclosure, visibility and HIV prevention and care, and the relationships between these groups and households of people living with HIV (see Supplementary files for topic guides). Data were systematically classified and organized by major themes and concepts [31] relating to collective efficacy and resistance to stigma, and the outcomes of these; factors that perpetuate stigma; and activities through which people living with HIV contribute to de-stigmatizing HIV.

### Ethical considerations

The study was approved by the Science and Ethics Committees of the Uganda Virus Research Institute and the Uganda National Council for Science and Technology. All personally identifiable information was deleted and data were held in a secure, password-protected computer at all times.

## Results

### Collective efficacy and resistance to stigma

In this study, challenging stigma transcended individual experiences and united people living with HIV in a process of social renegotiation. They sought to empower themselves and change their collective standing in the community. Challenging stigma transitioned from the individual to the collective domain.

People living with HIV wanted to mobilise so that they could come together and fight stigma and discrimination. (Focus group discussion, household members of people living with HIV, Jinja)

What motivated me to join this group was because we were isolated and stigma was too much in the community. (Focus group discussion, people living with HIV, Mbale)

Findings also suggest that increased interaction between people openly living with HIV and other community members through testimonials and other forms of interaction may have contributed to the perceived decline in stigma by demystifying HIV, as suggested by Brown *et al*. [21].

It has reduced because of the interaction between group members and community people. (Interview, male key informant, Jinja)

Involvement of people living with HIV in income-generating activities (within the Networks project) offered an opportunity for them to interact with their communities. This was particularly important given the relationship between poverty and HIV-related stigma in this setting, and more generally in sub-Saharan Africa [16].

Their success in … animal rearing and vegetable growing encouraged other community people to come and learn from the group, thereby increasing interaction between the community and the group members. (Interview, man living with HIV, Jinja)

People living with HIV who were successful in income-generating activities were no longer perceived as draining community resources, but as making a contribution instead, which underpins the material symbolism of HIV stigma [16].

Nowadays people in the community have realised the importance and usefulness of people living with HIV. They appreciate the role of the groups. This has reduced stigma. (Interview, man living with HIV, Jinja)

When the community members see the work we are doing in our groups, yet they didn't initially think we were capable of doing it, they start believing and having confidence in us. (Interview, woman living with HIV, Jinja)

Frequent notions emerged of the ways in which groups of people living with HIV increased their social capital through enhanced social inclusion and cohesion with their communities. This was determined by the contribution that the groups were perceived to be making, hence their “usefulness” to the larger community. Thus, being economically well-off appeared to cushion people living with HIV from being stigmatized, especially men.

I was not stigmatised or discriminated [against] because I was doing well financially and supporting my family ably. (Interview, man living with HIV, Jinja)

Not surprisingly, collective resistance was shaped by important factors driving stigma and self-stigma (feelings of shame, guilt and self-blame), including gender, family relationships and (as noted above) material wellbeing. Groups of people living with HIV responded to these factors either *directly*, for instance, by engaging in income generation to counter poverty, or *indirectly*, for instance, by proving a social space in which the impact of gender as a driver of stigma could be countered through peer support. This was particularly relevant given that social norms relating to men's role in society often contributed to self-stigma. Our study showed that it was men who had most difficulty in joining groups.

As men, we are [expected] to take care of our families. But because of poor health and stigma, we are unable to fulfil these family obligations. I had a lot of self-stigma and needed to join people with whom I could share the problem. (Focus group discussion, people living with HIV, Jinja)

There were many [people living with HIV] who were in hiding, especially men. Positive Men's Union encouraged them to come out. Men have been poor to join groups but this [group] will attract them more. (Interview, female key informant, Jinja)

Once mobilized, people living with HIV became involved in a number of activities that they saw as having an impact either on the level of stigma or on the way in which members coped with stigma (Table 2).

**Table 2:** Approaches and activities employed by groups of people living with HIV to counter HIV stigma

Approach	Illustrative quote
Peer support and counselling	*We needed to come together so that we could mobilise other people living with HIV in the communities, so that we could discuss and counsel one another to cope with stigma*. (Interview, woman living with HIV, Jinja)
	*The group members also go and reach out to people living with HIV in households who are facing problems like stigma and discrimination; support those on treatment to adhere to it; and also check on the general hygiene in the home*. (Focus group discussion, household members of people living with HIV, Jinja)
Community education and sensitization	*We have a drama group that goes around mobilising and sensitising people to create awareness*. (Focus group discussion, household members of people living with HIV, Jinja)
	*They also help bridge gaps of knowledge and clear myths that people have about HIV to reduce stigma*. (Interview, male key informant, Jinja)
	*The group has helped educate us and the community on issues like why test and how to overcome stigma and get self-confidence*. (Interview, male key informant, Mbale)
Media and printed information	*They are in [a drama group that] prepares songs [and] plays on HIV topics like [prevention of mother-to-child transmission] and the use of [antiretrovirals] and [their] benefits, and also on stigma and discrimination*. (Focus group discussion – household members of people living with HIV, Jinja)
	*We even talk on the radio and tell people we are … living with HIV*. (Interview, female key informant, Jinja)
Public testimonials and role modelling	*We also encourage giving of testimonies by people living with HIV in public*. (Focus group discussion – household members of people living with HIV, Jinja)
	*Public disclosure enabled me to reach out to others, to sensitise and educate them about HIV and to change people's attitudes towards people living with HIV*. (Interview, man living with HIV, Jinja)
	*They see me as an example and role model to copy from*. (Focus group discussion, people living with HIV, Jinja)

### Outcomes of collective efficacy and resistance

According to some study participants, groups’ activities had positive impacts on both self-stigma and stigma in the community.

Stigma amongst ourselves has reduced. There were members who had self-stigma, [but] today they are able to move out and talk about themselves. (Focus group discussion, people living with HIV, Jinja)

These groups have had an impact on communities’ attitudes towards people living with HIV. This has brought down the level of stigma and discrimination. (Interview, female key informant, Mbale)

Study participants reported that false beliefs regarding HIV were diminishing in the community.

They no longer think HIV is due to witchcraft because of an improved health-seeking culture, rather than going to shrines. (Interview, male key informant, Mbale)

While study participants reported that HIV stigma in their communities had generally declined over time, they believed it remained a powerful force in the lives of people living with HIV, even at the household level.

One of our members died recently as a result of being discriminated [against] and neglected by her family, who isolated her and failed to remind her to take her drugs. (Interview, man living with HIV, Jinja)

In addition, groups did not always have a positive impact on stigma. There were instances, especially initially, where association with groups was stigmatizing.

Many people feared coming to us openly, thinking that when others see them with us, they will be branded having HIV. (Interview, man living with HIV, Mbale)

## Discussion

Contrary to assertions that stigma may no longer be relevant in the face of a mature HIV epidemic and widespread antiretroviral access [13, 20], our study found that stigma remains a concern among people living with HIV in Uganda, where antiretroviral coverage is estimated to be between 52 and 81% [32]. We argue that our study captures a dynamic period in which stigma has started to diminish but has not yet been fully eliminated in the study districts. A recent study in Uganda showed that the impact of antiretroviral therapy on stigma is most marked during the first two years of treatment, after which its effect on stigma declines significantly [33]. This could account for the apparent paradox that stigma is both in decline and yet persistent in our study setting. This resonates with the traditionally held view that stigma is dynamic [12], and as such it could persist or even increase in the context of wider availability of antiretroviral therapy, as demonstrated in recent studies from Botswana [19] and South Africa [34].

An important finding from our study relates to how groups of people living with HIV can contribute to protecting their members from HIV stigma while at the same time de-stigmatizing HIV in their communities. Our study demonstrates that groups of people living with HIV can directly address factors known to influence HIV stigma, such as poverty [16], through collective participation in livelihood activities that would otherwise be difficult to accomplish individually, or through collective resistance by challenging stigma publicly. In our study, the collective activities of these groups (for instance, drama and income generation) provided practical skills to cope with external stigma, and confidence to overcome self-stigma. This pooling of labour and resources is a distinctive advantage of a “group” approach [35].

Our findings build and expand on the conceptual framework of effective approaches for reducing HIV stigma by Brown *et al*. [21]. This framework suggests that a high level of interaction and proximity between people with HIV and their communities demystifies HIV and reduces stigma [21]. While support groups of people living with HIV have been known to exist elsewhere [36], what was different about the groups in this study was how they were meaningfully involved not just in *receiving* but also in *providing* HIV services [28], as shown in Table 2 and in the intervention section of this paper. This provided them greater visibility and opportunities to interact with their communities, and empowered them to educate their communities and change their stigmatizing values. In that sense, they became agents of social change, as described by Parker and Aggleton [37]: they took active control of their health by collectively resisting factors undermining it. They also leveraged social capital to bridge their acceptability within their communities [38] by engaging in what were seen as “useful” activities, such as income generation and provision of HIV services.

These findings reinforce suggestions by Pulerwitz *et al*. [39] that engaging people living with HIV in programmes could be an effective strategy to reduce HIV stigma. This transformative social and economic participation of people living with HIV as a strategy to counter stigma is supported by evidence from India, Tanzania and Zambia showing that collective efficacy or resistance can improve the ability of marginalized groups to change their situation. Examples of this include sex workers confronting frequent arrests [40] and adolescents with HIV demanding services appropriate to their needs [41, 42].

This is not to suggest that groups of people living with HIV are sufficient alone to eliminate stigma. Rather, multiple approaches are required. Our study confirms that groups of people living with HIV in the two study districts were making a valuable contribution towards reducing stigma via collective efficacy – in effect, a demand-side initiative. However, this should be accompanied by other, supply-side interventions, such as sensitization training for teachers, health service providers, employers, law enforcement personnel, religious leaders and others, for an effective multi-sectoral mitigation of HIV stigma [8, 43, 44]. In addition, the environment in which such groups operate could determine their impact. Our study was conducted in Uganda, which has been hailed as a success in its response to HIV partly due to an “open general environment which allows open discussions surrounding HIV” [45, p. 2]. This may have created an enabling environment for the groups to have an impact.

While our findings suggest that community-based groups of people living with HIV could enable their members to better cope with stigma, the limitations of such groups should be noted. For instance, there is the risk of further alienating groups of people with HIV from their communities through the creation of new notions of social citizenship [46] that could emerge from their collective identity and shared responsibility to sensitize and ‘educate’ others. Roopnaraine *et al*. [35, p. 649] warn that the “problem of stigma inherent in joining groups defined by HIV status” must be carefully balanced with the benefits of such groups.

### Implications for programming and research

These findings have important implications for programming and research. First, they provide a basis for extending current approaches to reducing stigma beyond interventions that seek to increase the resilience and coping mechanisms of *individuals* to those that strengthen the capacity of groups to *collectively* challenge stigma. This could enable people living with HIV who participate in networked groups to leverage social capital, cope with stigma, participate in HIV programmes and enhance their uptake of HIV services [28, 37]. Our findings also inform gender constructs around HIV stigma. Wyrod [47] argues that the inextricable link between the experiences of men with regard to HIV stigma and conceptions of masculinity highlights challenges to, *and opportunities* for, addressing stigma. In our study, societal expectations of men contributed in distinctive ways to their experiences of HIV stigma, suggesting that as HIV programmes in sub-Saharan Africa strive to engage men in HIV care [47, 48], interventions to address HIV stigma should be gender sensitive. This is particularly relevant considering that men in our study were reluctant to join groups, which often prompted creation of men-only groups such as Positive Men's Union (*see* Table 1).

### Limitations

The qualitative nature of our data restricts generalizability, although the study does provide important in-depth insight into the potential of engaging people living with HIV as agents of change in challenging stigma. Our findings relate to two of the 40 districts in which the intervention was implemented, further limiting generalizability of our findings to the remaining districts, especially considering that experiences of stigma could differ between urban and rural contexts. However, our findings could complement those from other stigma studies and stigma index surveys, (for example those that were being conducted by the National Forum for Networks of people living with HIV in Uganda at the time of writing this manuscript), in informing future interventions. Finally, our data did not capture information relating to the process and challenges of setting up groups, which could be valuable in interpreting our findings. Future research should explore long-term impacts of the collective activities of groups of people living with HIV.

## Conclusions

Meaningful engagement of people living with HIV can contribute to interventions to mitigate HIV stigma. Antiretroviral therapy and group-based approaches are opening up new avenues for the collective participation of people living with HIV to change community attitudes towards HIV. Current approaches to reducing stigma should be extended beyond interventions that seek to increase the resilience and coping mechanisms of individuals, to those that build the capacity of groups to collectively challenge stigma.
